# Are adrenergic α1- antagonists beneficial for the access of retrograde ureteral access sheath or semi-rigid ureteroscope access? A systematic review and meta-analysis

**DOI:** 10.3389/fsurg.2022.1055904

**Published:** 2023-01-05

**Authors:** Qibo Hu, Chi Yuan, Sikui Shen, Zhongyu Jian, Xi Jin, Yucheng Ma, Hong Li, Kunjie Wang

**Affiliations:** Department of Urology, Institute of Urology (Laboratory of Reconstructive Urology), West China Hospital, Sichuan University, Chengdu, China

**Keywords:** meta-analysis, adrenergic α1- antagonists, retrograde ureteral surgery, urinary stone, urolithiasis

## Abstract

**Introduction:**

To evaluate the clinical benefit of preoperative adrenergic α1-antagonist therapy in the management of upper urinary calculi.

**Materials and methods:**

Publications were searched for The Cochrane Central Register of Controlled Trials, EMBASE, and MEDLINE until 1 March 2022 that related to the adrenergic α1- antagonist intake as adjunctive therapy before retrograde surgery. Dichotomous data were reported with risk ratios (RR) with 95% confidence intervals (CIs) and the continuous data were reported with mean difference (MD) with 95% CIs

**Results:**

There were nine studies with 867 patients included in this meta-analysis. Preoperative adrenergic α1- antagonists could significantly elevate the compared with the placebo. Higher successful access rate to the stone was found in patients who received preoperative adrenergic α1- antagonists than those who received the placebo (RR 1.24; 95% CI 1.17–1.33). Besides, the application of preoperative adrenergic α1- antagonists can also elevate 4th-week stone-free rate (RR 1.20; 95% CI 1.12–1.28), decrease postoperative analgesia (RR 0.30;95% CI 0.20–0.46) and result in a lower risk of overall complications (RR 0.38; 95% CI 0.24–0.61).

**Conclusion:**

Preoperative adjunctive adrenergic α1- antagonist therapy is effective and safe in the management of retrograde surgery with a higher successful access rate and lower risk of severe complications.

## 1. Introduction

As one of the most common urological diseases, urolithiasis has a high incidence varied from 1.7% to 14.8%, depending on various factors like geography, climate and gene, etc. (1, 2). It is even reported that in the past 20 years, the incidence had increased by more than 37% in some regions (3, 4). With the development of Technology, including the miniaturization of endoscopes, the improvement of deflection mechanism, and upgraded optical quality and tools have resulted in the increased use of ureterorenoscopy (URS) for urolithiasis. Retrograde intrarenal stone surgery (RIRS) achieves major technological progress in lithotripsy efficiency. Recently, a systematic review indicated that renal stones >2 cm demonstrated a stone-free rate (SFR) of 91% with 1.45 procedures/patient (5).

However, routine pre-stenting before URS is still inconclusive. Pre-stenting implantation can promote the ureteroscopic management of urolithiasis, increase the SFR, and decrease perioperative complications such as ureteral perforation and avulsion, but it also brings the risk of urinary tract infection (UTI), lower urinary tract discomfort (ureteral stent-related symptoms) and additional financial burden to the patient (6, 7). EAU guidelines recommend that patients with increased risk of complications like ureteral trauma, perforation, UTIs, etc, should be inserted with stents to avoid stressful emergencies (8). Therefore, a simpler and more effective preoperative preparation (such as oral medication) makes a lot of sense.

Adrenergic α1- antagonist (Alpha-1 blocker, AB) medications have been proved to increase the rate of stone expulsion for stones larger than 5 mm in the distal ureter after extracorporeal shock wave lithotripsy (SWL) and ureteroscopy (9, 10). These effects were due to the inhibition of alpha-1 adrenergic receptor can cause relaxation of ureteral smooth muscle and thereby reduce the intensity and frequency of physiologic ureteral peristalsis (11). Some surgeons supposed that AB medications might be helpful during ureteroscopic procedures because of their relaxing effect on the ureter *in vivo* (12). New research shows that oral adrenergic alpha-antagonists before URS might be beneficial to increase the successful access rate of RIRS, increasing 4th week SFR after URS, decreasing the risk of intra-operative ureteral dilatation, and protecting against ureteral injury (13–15). However, only a few research with high quality have assessed the outcome of AB application on ureteroscopic access in patients undergoing RIRS, and the results remained inconclusive. Therefore, this systematic review and meta-analysis aimed to illuminate the effects of ABs treatment before ureteroscopy on the success of RIRS.

## 2. Method

### 2.1. Search strategy

We performed and reported the analysis according to the general guidelines recommended by the Primary Reporting Items for Systematic Reviews and Meta-analyses (PRISMA) statement, and registered this study on PROSPERO (CRD42022325259) (16). Inclusion criteria were established before searching. We used the Cochrane Central Register of Controlled Trials (*via* Wiley), Embase, and Medline (*via* PubMed) databases until 1 March 2022 to search for all published studies evaluating the successful access rate of patients undergoing RIRS after AB therapy.

The PICO strategy was developed in order to perform an accurate search strategy. Population were patients received retrograde intrarenal stone surgery; intervention studied was preoperative adrenergic α1- antagonist therapy; comparison was no preoperative adrenergic α1- antagonist therapy; The primary outcome was the successful access rate and the secondary outcomes were 4th week Stone-free-rate, operation time, postoperative analgesia, complications.

The followings search strings were applied: (((silodosin[Title/Abstract]) OR (alfuzosin[Title/Abstract]) OR (tamsulosin[Title/Abstract]) OR (doxazosin[Title/Abstract]) OR (terazosin[Title/Abstract]) OR (naftopidil [Title/Abstract]) OR (Adrenergic alpha-Antagonists[Title/Abstract]) OR (α-Adrenergic Antagonists[Title/Abstract])) AND ((ureter[Title/Abstract]) OR (ureters[Title/Abstract]) OR (ureteral[Title/Abstract])) AND ((access[Title/Abstract]) OR (enter[Title/Abstract]) OR (entry[Title/Abstract]) OR (pass[Title/Abstract]) OR (forward[Title/Abstract]))).

The above shows the strategy we used in PubMed as an example. The controlled vocabulary (such as MeSH in PubMed and EMTREE in Embase) and entry terms were used when possible. The precise strategy was tailored to accommodate each database's features, The other search strategies are available on request.

Publications that met the following criteria were included: reporting original research; English language; human studies; enrolling undergoing RIRS patients; and reporting successful access rate after treatment with an AB. Reference list in relevant articles and reviews were also screened for additional studies. Abstracts (with no subsequent full-text publications) and unpublished studies were excluded. Two authors (QH and CY) reviewed the records separately to select relevant publications, with any discrepancies resolved by open discussion. The quality of the randomized controlled trials (RCTs) was estimated using the Modified Jadad score and RoB 2 (Version 2 of the Cochrane tool for assessing risk of bias in randomised trial), and the retrospective studies were evaluated using the Newcastle-Ottawa Quality Assessment Scale of cohort studies (17, 18).

### 2.2. Data extraction

The following data were extracted from the studies included: study and publication year; country; type of AB used and comparison; study design; Quality assessment of the study; sample size; the number of patients at baseline; subtype of RIRS; the size of the equipment; access rate; operative time; 4th week SFR; Any complications. An online calculator was also applied (https://www.math.hkbu.edu.hk/∼tongt/papers/median2mean.html) to estimate the sample mean and standard deviation from the sample size, median, range, and/or interquartile range (19).

### 2.3. Outcomes of interest

The primary outcome was the successful access rate. 4th week SFR; operation time; postoperative analgesia and complications were set as the secondary outcomes.

### 2.4. Statistical analysis

Review Manager Software 5.3 (The Cochrane Collaboration, Nordic Cochrane Centre, Copenhagen, Denmark) was used to perform the analysis. Dichotomous variables are described as Risk Ratio (RR), Mantel-Haenszel weight, and 95% confidence intervals (CIs) for each study. For continuous variables, mean difference (MD) estimate, standard error, inverse-variance weight, and 95% CIs for each study were reported. Generic Inverse Variance variables are reported as Risk Difference, inverse-variance weight, and 95% CIs for each study. Statistical pooling for MD estimates was performed according to a Random Effects model with generic inverse-variance weighting, computing estimates with 95% CI. Study bias was appraised by graphical inspection of funnel plots. Hypothesis testing for superiority was set at a two-tailed level of 0.05. Hypothesis testing for statistical homogeneity was set at a two-tailed level of 0.10 and was based on the Cochran *Q* test, with *I*^2^ values of 25%, 50%, and 75% representing mild, moderate, and extensive statistical inconsistency, respectively (20). If *I*^2^ values ≥50%, we preferred the random effects model to the analysis, otherwise we used the fixed effect model. Forest plots were generated to demonstrate the successful access rate (primary outcome) during RIRS with AB medication versus placebo. Subgroup analyses were conducted according to the study design, age bracket of the patients, access subtype, and classification of AB.

## 3. Results

### 3.1. Study characteristics

**Figure 1:**
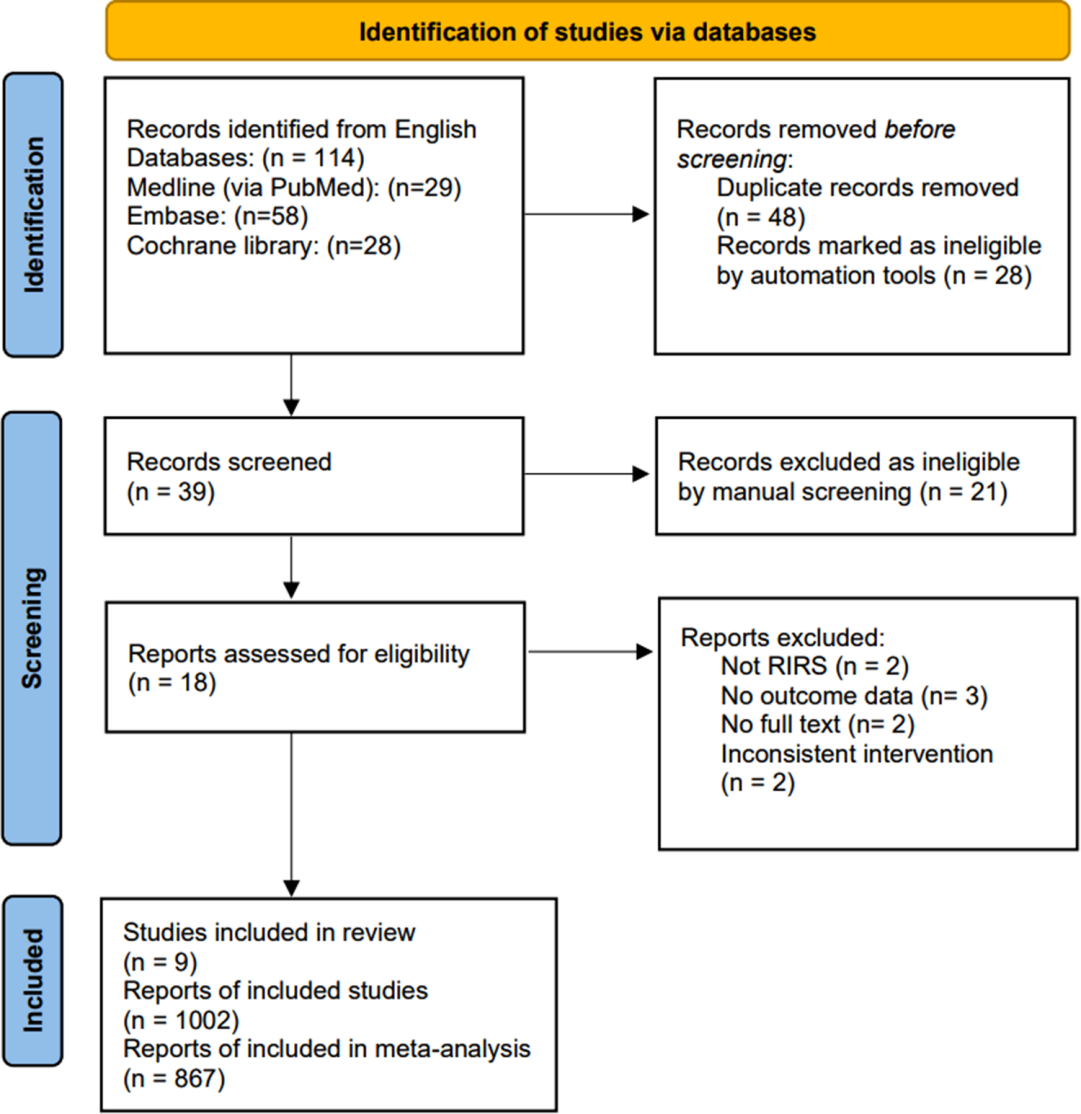
PRISMA 2020 flow diagram m for new systematic reviews which included searches of databases and registers only. *Consider, if feasible to do so, reporting the number of records identified from each database or register searched (rather than the total number across all databases/registers). **If automation tools were used, indicate how many records were excluded by a human and how many were excluded by automation tools.

The study selection process is presented in Figure 1. Initially, there were 114 publications revealed after database searches up to March 2022. Among these, 48 were removed due to duplicated series. Further, 49 were excluded according to the title or abstract by automation tools and manual screening. Finally, nine studies with 867 patients were included in the meta-analysis (12, 13, 15, 21–26). Specifically, six studies were RCTs, and three were retrospective studies. We used Modified Jadad Score and RoB 2 to assess the quality of RCTs, while retrospective studies were assessed by Newcastle-Ottawa Scale (specific scoring refers to Supplementary Material). All included studies were assessed as low risk of bias. The characteristics of the nine studies are presented in Table 1. The funnel plot suggesting that publication bias was present in Supplementary Material.

**Table 1:** Characteristics of the studies included in the review.

Study	Country	Comparison	Lasting time before surgery	Total No. of pts	Design	Quality assessment	Age brackets	Subtype	Size (Fr)	Age (yr), mean (SD)	Access rate (%)	Operative time, (min)	4th week stone-free rate (%)	Complications (%)
Ahmed (2016)	Saudi Arabia	0.4 mg tamsulosin vs. placebo	7 days	165	RCT	6/7	Adults	Semi-rigid access	7.5	36.7 (11.1)	93.8 (76/81) vs. 82.1 (69/84)	43.4 (12.3) vs. 49.6 (13.6)	91.4 vs. 79.8	17.3 vs. 38.1
Aydin (2017)	Turkey	8 mg silodosin 1day vs. 8 mg silodosin 3day vs. placebo	3 days	147	RCT	6/7	Adults	Semi-rigid access	7/7.5	38.1 (9.6) vs. 44.3 (12.6) vs. 38.7 (10.9)	78.0 (39/50) vs. 95.7 (45/47) vs. 76.0 (38/50)	29.6 (7.8) vs. 30.0 (9.0) vs. 32.6 (13.4)	74.0 vs. 93.6 vs. 74.0	4.0 vs. 0 vs. 12.0
Bhattar (2017)	India	8 mg silodosin vs. 10 mg tadalafil vs. placebo	14 days	67	RCT	6/7	Adults	Semi-rigid access	8/9.8	35.5 (11.0) vs. 42.8 (14.0) vs. 33.2 (10.7)	73.9 (17/23) vs. 60.9 (14/23) vs. 28.6 (6/21)	35.2 (5.6) vs. 34.9 (5.0) vs. 41.1 (2.5)	NR	17.4 vs. 26.1 vs. 52.5 (Hematuria) 13.0 vs. 17.4 vs. 38.1 (Mucosal injury) 17.4 vs. 13.0 vs. 28.6 (Fever)
Mohey (2018)	Egypt	8 mg silodosin vs. placebo	10 days	127	RCT	7/7	Adults	Semi-rigid access	9.5	38.3 (9.4) vs. 39.7 (9.5)	96.8 (60/62) vs. 80.0 (52/65)	41.61 (4.67) vs. 46.85 (4.6)	94.6 vs. 75.4	6.4 vs. 20.0
Bayar (2019)	Turkey	0.4 mg tamsulosin vs. 50 mg mirabegron vs. placebo	7 days	186	RCT	6/7	Adults	Semi-rigid access	7.5	42.1 (11.4) vs. 42.8 (13.5) vs. 39.0 (14.6)	96.7 (59/61) vs. 95.2 (59/62) vs. 81.0 (51/63)	33 (9.4) vs. 33 (8.3) vs. 30.9 (6.6)	90.2 vs. 95.2 vs. 77.8	1.6 vs. 1.6 vs. 3.2
Demir (2022)	Turkey	0.4 mg tamsulosin vs. placebo	7 days	137	RCT	3/7	Adults	Semi-rigid access	8/9.8	45.8 (16.8) vs. 45.8 (14.0)	94.0 (63/67) vs. 81.4 (57/70)	48.80 (14.19) vs. 55.50 (11.95)	88.0 vs. 72.9	9.0 vs. 22.9
Erturhan (2019)	Turkey	0.4 mg tamsulosin vs. placebo	14 days	48	Retrospective	8/9	Adults	UAS access	9.5/11.5	40.5 (12.3) vs. 38.5 (9.5)	65.2 (15/23) vs. 44.0 (11/25)	54.4 (6.03) vs. 58.36 (6.54)	93.3 vs. 90.9	6.6 vs. 9.1 (Fever) 13.2 vs. 9.1 (UTI) 0 vs. 18.2 (Steinstrasse) 4.3 vs. 4.0 (Sepsis)
Morley (2020)	USA	0.4 mg tamsulosin vs. placebo	>2 days	76	Retrospective	7/9	Children	Semi-rigid access/UAS access	4.5 (semi-rigid) 9.5 (UAS)	12.2 (3.5) vs. 11.7 (3.3)	88.0 (44/50) vs. 65.4 (17/26) (total) 87.5 (21/24) vs. 20.0 (1/5) (Semi-rigid) 88.5 (23/26) vs. 76.2 (16/21) (UAS)	NR	NR	NR
McGee (2021)	USA	0.4 mg tamsulosin vs. placebo	>7 days	49	Retrospective	8/9	Children	UAS access	7.95	13.0 (3.8) vs. 13.7 (4.3)	61.5 (8/13) vs. 38.9 (14/36)	40.5 (26.2) vs. 36.1 (16.0)	75.0 vs. 50.0	NR

Total No. of pts, total number of patients; Fr, French; yr, year; SD, standard deviation. Quality assessment of the included studies: RCTs were assessed by Modified Jadad Score and Retrospective studies were valued by Newcastle-Ottawa Scale of cohort study. Age (yr), mean (SD): We unified the format of the data by estimating the sample mean and standard deviation from the sample size, median, range and/or interquartile range. Stone-free rate: Computed tomography scans were followed up after treatment and compared with those before surgery to confirm the proportion of stone-free patients to all subjects. Complications (%): We counted overall complications (per-person) if they were reported in the study, otherwise the total number of different types of complications (per-time) were calculated.

### 3.2. Primary outcome

**Figure 2:**
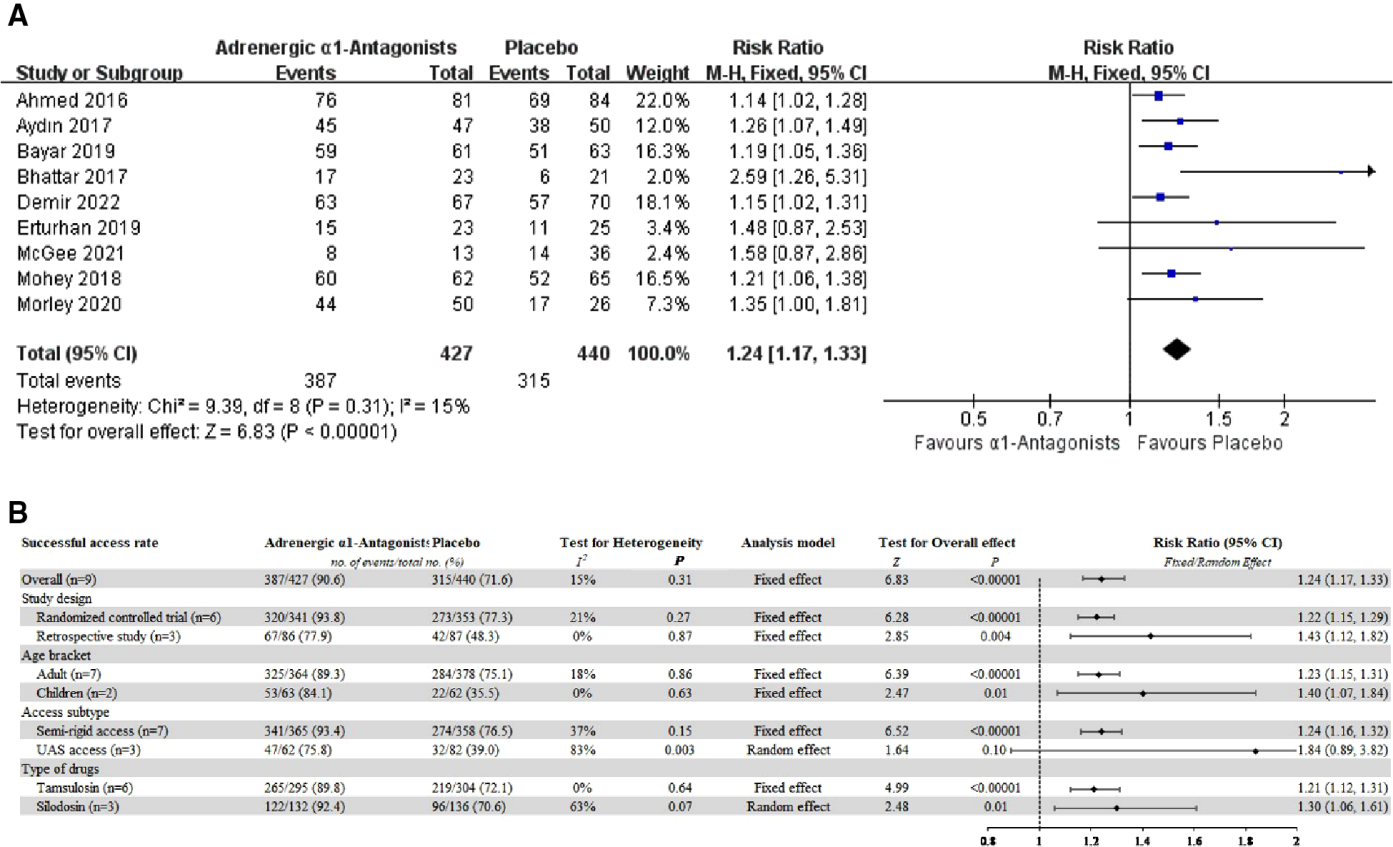
(**A**) Forest plot for overall successful access rate of RIRS. (**B**) Forest plot for subgroup analysis.

The overall successful access rate of RIRS and the subgroup analysis were displayed in Figures 2A,B.

Compared with placebos, patients who received AB medications had a significantly higher first access rate of RIRS (9 studies, 867 patients: pooled RR 1.24, 95% CI 1.17–1.33; Chi^2^ = 9.39; *I*^2^ = 15%; *P* < 0.00001; mild statistical inconsistency).

Both RCT (6 studies, 694 patients: pooled RR 1.22, 95% CI 1.15–1.29; Chi^2^ = 6.34; *I*^2^ = 21%; *P* < 0.00001; mild statistical inconsistency) and retrospective study (3 studies, 173 patients: pooled RR 1.43, 95% CI 1.12–1.82; Chi^2^ = 0.28; *I*^2^ = 0%; *P* = 0.004; mild statistical inconsistency) have confirmed that preoperative AB medication can improve the success rate of retrograde semi-rigid access and the placement of ureteral access sheath.

The results of subgroup analysis showed that both adults (7 studies, 742 patients: pooled RR 1.23, 95% CI 1.15–1.31; Chi^2^ = 7.34; *I*^2^ = 18%; *P* < 0.00001; mild statistical inconsistency) and children (2 studies, 125 patients: pooled RR 1.40, 95% CI 1.07–1.84; Chi^2^ = 0.23; *I*^2^ = 0%; *P* = 0.01; mild statistical inconsistency) could benefit from preoperative AB medication.

AB medication had inconsistent results for different RIRS, the successful access rate of semi-rigid had significant differences in patients taking adrenergic α1-antagonists preoperatively (7 studies, 723 patients: pooled RR 1.24, 95% CI 1.16–1.32; Chi^2^ = 9.46; *I*^2^ = 37%; *P* < 0.00001; moderate statistical inconsistency). However, the ureteric access sheath (UAS) access rate with or without AB medication was no significant difference between the data (3 studies, 144 patients: pooled RR 1.84, 95% CI 0.89–3.82; Chi^2^ = 11.49; *I*^2^ = 83%; *P* = 0.10; extensive statistical inconsistency).

Different types of adrenergic α1-antagonists did not affect the overall successful access rate of RIRS, both tamsulosin (6 studies, 599 patients: pooled RR 1.21, 95% CI 1.12–1.31; Chi^2^ = 3.40; *I*^2^ = 0%; *P* < 0.00001; mild statistical inconsistency) and silodosin (3 studies, 268 patients: pooled RR 1.30, 95% CI 1.06–1.61; Chi^2^ = 5.45; *I*^2^ = 63%; *P* = 0.01; extensive statistical inconsistency) were effective.

### 3.3. Secondary outcome

We set 4th week SFR; operation time; postoperative analgesia and postoperative complications as the secondary outcome.

#### 3.3.1. The fourth-week stone-free rate

**Figure 3:**
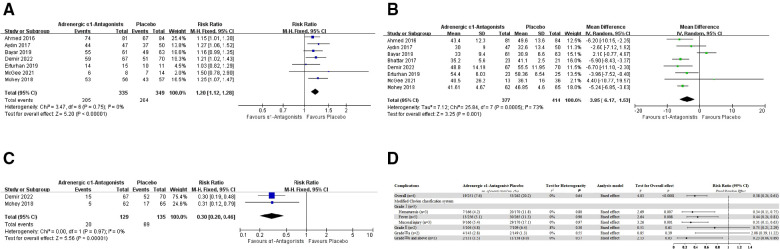
(**A**) Forest plot for 4th week stone free rate. (**B**) Forest plot for operation time. (**C**) Forest plot for postoperative analgesia. (**D**) Forest plot for postoperative complications.

Seven studies contributed to the analysis of the 4th week SFR. Compared with placebos, preoperative oral adrenergic α1-antagonists intake helps to improve the 4th week SFR (pooled RR 1.20, 95% CI 1.12–1.28; Chi^2^ = 3.47; *I*^2^ = 0%; *P* < 0.00001; mild statistical inconsistency) (Figure 3A).

#### 3.3.2. Operation time

Eight studies contribute to the analysis of operation time. We found heterogeneity among these trials (*I*^2^ = 73%; *P* = 0.0005), thus we chose a random-effects model for this analysis. The AB medication group can probably reduce the operation time compared with the placebo group (pooled MD −3.85, 95% CI −6.17 to −1.53; Chi^2^ = 25.84; *I*^2^ = 73%; extensive statistical inconsistency) (Figure 3B).

#### 3.3.3. Postoperative analgesia

Only two studies reported postoperative analgesia in patients after RIRS. Compared with the placebo group, the proportion of patients taking adrenergic α1-antagonists preoperatively requiring postoperative medication analgesia was significantly reduced (pooled RR 0.30, 95% CI 0.20–0.46; Chi^2^ = 0.00; *I*^2^ = 0%; *P* < 0.00001; mild statistical inconsistency) (Figure 3C).

#### 3.3.4. Postoperative complications

In general, seven studies reported postoperative complications in patients, and all these were in adults. Overall complications were defined as “total complications” only if the number of all complications was specifically reported in the original study. We subjectively graded the complications in the original article according to the modified Clavien-Dindo classification system (MCCS) (27). Subgroup analysis was performed for common complications (such as hematuria, fever, and mucosal injury).

Four studies contribute to the overall complications analysis. Compared with the placebo group, the adjunctive ABs therapy was related to a considerably lower incidence of overall complications (pooled RR 0.38, 95% CI 0.24–0.61; Chi^2^ = 1.68; *I*^2^ = 0%; *P* < 0.0001; mild statistical inconsistency) (Figure 3D). More specifically, the above differences were mainly due to the complications with an MCCS score of Grade I. 2160;. Compared with the placebo group, the incidence of postoperative hematuresis (three studies, *n* = 336; 166 in the AB medication group and 170 in the placebo group, pooled RR 0.34, 95% CI 0.15–0.75; Chi^2^ = 0.45; *I*^2^ = 0%; *P* = 0.007; mild statistical inconsistency), fever (five studies, *n* = 521; 256 in the AB medication group and 265 in the placebo group, pooled RR 0.44, 95% CI 0.24–0.81; Chi^2^ = 1.09; *I*^2^ = 0%; *P* = 0.008; mild statistical inconsistency), and mild mucosal injury (three studies, *n* = 336; 166 in the AB medication group and 170 in the placebo group, pooled RR 0.31, 95% CI 0.15–0.63; Chi^2^ = 0.05; *I*^2^ = 0%; *P* = 0.001; mild statistical inconsistency) were all significantly lower in the AB medication group.

There was no significant difference in Grade II and Grade IIIa between the adjunctive AB medication and placebo group. Grade IIIb and above was defined as requiring general anesthesia intervention or life-threatening complications, including ureteral perforation, formation of a false lumen, mucosal hemorrhage requiring the operation to end, Steinstrasse and sepsis after RIRS. Compared with the placebo group, preoperative oral adrenergic α1-antagonists intake helps to reduce the incidence of those serious complications (pooled RR 0.23, 95% CI 0.06–0.89; Chi^2^ = 1.11; *I*^2^ = 0%; *P* = 0.03; mild statistical inconsistency) (Figure 3D).

## 4. Discussion

In this meta-analysis, we found that preoperative adrenergic α1- antagonists was beneficial to the successful access rate of RIRS. Furthermore, prior AB medication could also increase the 4th week SFR, speed up the process of retrograde surgery, decrease postoperative analgesia and reduce the incidence of some complications.

The α1-adrenergic receptors can be divided into 3 distinct subtypes: α1A, α1B, and α1D. The human ureter contains these receptors throughout its entire length, particularly the α1A and α1D subtypes, which are more densely located at the distal ureterovesical and ureterovesical junctions compared with the middle and proximal ureters (28). The stimulation of α1-adrenergic receptors has been proved to enhance ureteral contraction and increase its peristalsis (29). Therefore, selective α1A/α1D-adrenergic receptor blockers, such as tamsulosin and silodosin have already been used as an initial treatment for patients with ureteric stones <10 mm in size as Medical expulsive therapy (MET) and increase the spontaneous passage of stone fragments in the ureter after SWL and ureteroscopy (30, 31). In recent years, some scholars have speculated that preoperative AB medication intake may also be beneficial to RIRS (21, 22). Several RCTs and retrospective studies have reported favorable outcomes in adults or children with adjunctive AB medication before RIRS (23–25). So far, the efficacy of alpha-blocker application before routine ureteroscopy for upper urinary urolithiasis remains unclear and controversial.

In this study, we analyzed the efficacy and safety of AB medication before RIRS through meta-analysis to obtain a robust conclusion. To our knowledge, this is the first meta-analysis providing comprehensive insight into the effects of prior AB intake and the outcomes of RIRS. According to the study, patients who received AB medication had a significantly higher first access rate of RIRS. We further performed a subgroup analysis by study type, age bracket, the subtype of retrograde surgery, and different types of α1D-adrenergic receptor antagonists to reduce the clinical heterogeneity. Subgroup analysis showed that the conclusions above were credible and applicable to both adults and children. It should be noted that the two articles reporting the effect of AB medication on children's RIRS were all retrospective studies, so the level of evidence would be lower. We also conducted a subgroup analysis based on the initial insertion of a semi-rigid ureteroscope or UAS. The results showed that AB medication significantly improved the success rate of the semi-rigid ureteroscope forward, but there was no statistical significance for the placement of UAS. We speculated that the improvement effect of AB medication was closely related to the outer diameter of the implant, when the implant's outer diameter was greater than 10Fr, the improvement effect of AB drug treatment might be reduced. However, this did not mean that AB medication is not beneficial for the implantation of UAS. Several articles have reported that prior AB intake can reduce the insertion of shear force on the distal ureter during the forward of UAS, thus reducing intraoperative ureteral wall injury and postoperative pain in the patients (14, 32). In accordance with the subgroup analysis, both tamsulosin and silodosin were effective with minor side effects. Due to differences in pharmacokinetics, silodosin usually works after 3 days of oral use, while tamsulosin takes at least 4 days to a week (33, 34). In clinical practice, urologists can use them flexibly according to administration time and economic cost.

The adrenergic α1- antagonist can cause relaxation of the ureteric smooth muscles and dilatation of the ureteric lumen, especially the distal ureter and ureterovesical junction (29). Therefore, prior AB medication facilitates the search for the ureteral orifice during the operation and the smooth progress of retrograde catheterization, effectively reducing the operation time. The dilated ureteral lumen after the action of the AB medication is beneficial to the lithecbole of the residual stones and reduces the analgesic requirement during the process of stone removal. In the current meta-analysis, the adjunctive α1-blocker therapy was related to a significantly lower incidence of postoperative complications than the placebo group, primarily with an MCCS score of in Grade I and Grade IIIb and above. Drug-induced ureteral lumen expansion can effectively reduce hematuria, mild ureteral injury, and postoperative fever caused by lithotripsy and effectively reduce the incidence of serious surgical complications (ureteral perforation, etc.), suggesting that the AB medication resulted in safer ureteroscopic procedures. In addition, we conducted a separate meta-analysis of the successful access rate (Supplementary Material), with initial catheterization successful access rate of 0.92 (pooled risk difference 0.92, 95% CI 0.88–0.96; Chi^2^ = 23.93; *I*^2^ = 67%; *P* = 0.002; extensive statistical inconsistency) in AB medication group and only 0.66 (pooled risk difference 0.66, 95% CI 0.55–0.77; Chi^2^ = 59.79; *I*^2^ = 87%; *P* < 0.00001; extensive statistical inconsistency) in the placebo group. Several studies have reported that the successful access sheath insertion rate of the pre-stented patient was 92%–97% (35, 36). Despite the high heterogeneity of the single rate meta-analysis and the lack of high-level RCT studies between the prior AB medication and pre-stented, we believe that for the general population, the efficacy and safety of catheterization in the AB medication group do not make much inferior to that in the pre-stented group. These conclusions suggest whether we can use oral drugs as a more convenient way to replace routine stenting to ensure the efficiency and safety of RIRS for people with a low risk of postoperative complications.

Although there have been studies for the preoperative α-blockers for ureteroscopy, the focus of these studies has been on postoperative stone-free rate (37, 38). A key strength of this study is that we focused more on the primary success rate of retrograde surgery. For patients with ureteroscopy or ureteral access sheath cannot pass during surgery, blindly intraoperative ureteral dilatation would increase the probability of ureteral stricture, chosen intraoperative placement of the ureteral stent and elective surgery is safe and feasible, but that bring inconvenience to patients as well as the waste of medical resources. We believe that as ureteral lithotripsy becomes more mature and standardized, the first successful access rate of ureteroscopy is as important as the stone-free rate. In this study, we performed subgroup analyses according to age group, type of α-blocker, and size of ureteroscope, and included more high-quality studies, which further improved the quality and clinical value of the study.

However, our study has limitations. (1) Due to the limited number of relevant original studies, only nine studies (six RCTs and three retrospective studies) were included in this meta-analysis with relatively small sample size. (2) the intervention period with different adjunctive alpha-blockers before RIRS varied from 3 days to 1 week. (3) inconsistent size of the ureteroscope or UAS exists across the studies, ranging from 4.5 Fr to 11.5 Fr. (4) other information (postoperative double-J stent placement rates, SWL histories, and the use of the AB medication after the ureteroscopy) were incomplete. Additionally, the lack of unified inclusion criteria, different types of applied alpha-blockers and ureteroscope, the various locations of the ureteral stones, and the different age brackets of the patients may have resulted in bias. We reduce these deviations as much as possible by subgroup analysis and reclassifying the raw data. Further multicenter RCTs with high quality are warrant to provide more information on the application of adjunctive AB medication before RIRS for the treatment of upper urinary tract urolithiasis.

## 5. Conclusion

This meta-analysis provided evidence that preoperative adjunctive adrenergic α1- antagonist therapy was effective and safe in the management of RIRS. These findings suggest oral ABs may be as a more convenient way to replace routine stenting to increase the efficiency of RIRS without compromising safety outcomes.

## Data Availability

The original contributions presented in the study are included in the article/**Supplementary Material**, further inquiries can be directed to the corresponding author.
