# Supplementary material for: Are adrenergic α1- antagonists beneficial for the access of retrograde ureteral access sheath or semi-rigid ureteroscope access? A systematic review and meta-analysis
Source: Front Surg. 2023 Jan 5;9:1055904. doi: 10.3389/fsurg.2022.1055904 (PMC9849750; doi:10.3389/fsurg.2022.1055904)

1 Modified Jadad Score (7-point)

Study	Country	Items				Score
		Randomization	Concealment of allocation	Double blinding	Withdrawals and dropouts	
Ahmed (2016)	Saudi Arabia	2	2	1	1	6
Aydin (2017)	Turkey	2	1	2	1	6
Bhattar (2017)	India	2	2	1	1	6
Mohey (2018)	Egypt	2	2	2	1	7
Bayar (2019)	Turkey	2	2	1	1	6
Demir (2022)	Turkey	1	1	1	0	3

2 Risk of Bias 2 (RoB 2)

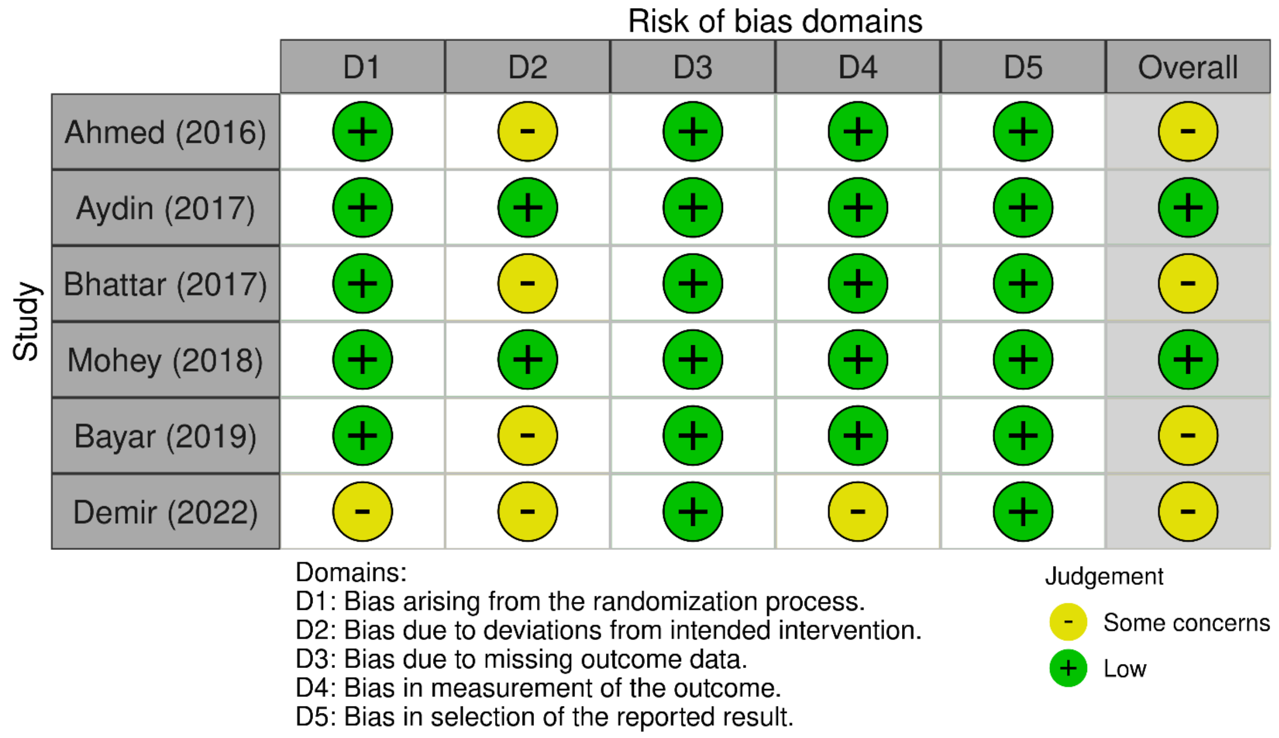

3 Newcastle-Ottawa Quality Assessment Scale Cohort Studies (9-point)

Study	Country	Items			Score
		Selection	Comparability	Outcome	
Erturhan (2019)	Turkey	3	2	3	8
Morley (2020)	USA	3	1	3	7
McGee (2021)	USA	3	2	3	8

4 Begg's funnel plot with pseudo 95% confidence limits

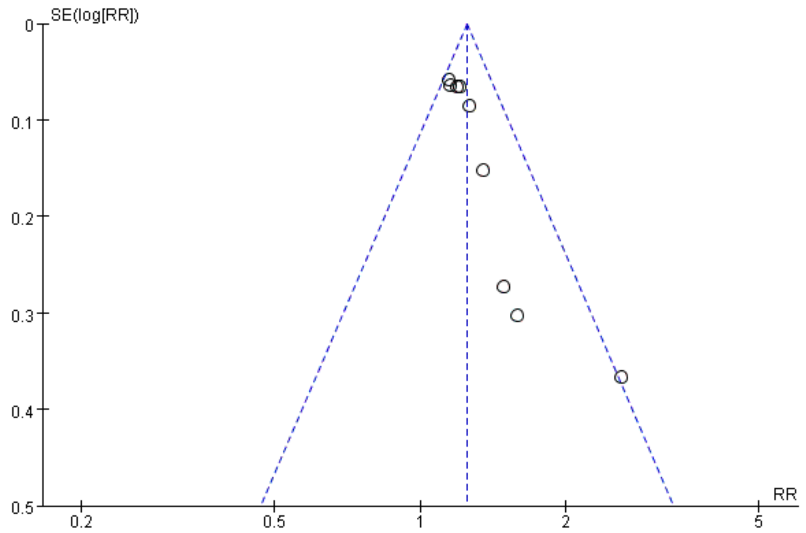

5 meta-analysis of the postoperative complications

	Adrenergic α1-Antagonists		Placebo			Risk Ratio
**Study or Subgroup**	**Events**	**Total**	**Events**	**Total**	**Weight**	**M-H, Fixed, 95% CI**
**1.1.1 Overall Complications**						
Ahmed 2016	14	81	32	84	60.0%	0.45 [0.26, 0.79]
Aydın 2017	0	47	6	50	12.0%	0.08 [0.00, 1.41]
Bayar 2019	1	61	2	63	3.8%	0.52 [0.05, 5.55]
Mohey 2018	4	62	13	65	24.2%	0.32 [0.11, 0.94]
**Subtotal (95% CI)**		**251**		**262**	**100.0%**	**0.38 [0.24, 0.61]**
Total events	19		53			
Heterogeneity: $Chi^2 = 1.68$, df = 3 (P = 0.64); $I^2 = 0\%$						
Test for overall effect: Z = 4.03 (P < 0.0001)						
**1.1.2 Grade Ⅰ Hematuresis**						
Ahmed 2016	2	81	4	84	19.3%	0.52 [0.10, 2.75]
Bhattar 2017	4	23	11	21	56.6%	0.33 [0.12, 0.88]
Mohey 2018	1	62	5	65	24.0%	0.21 [0.03, 1.74]
**Subtotal (95% CI)**		**166**		**170**	**100.0%**	**0.34 [0.15, 0.75]**
Total events	7		20			
Heterogeneity: $Chi^2 = 0.45$, df = 2 (P = 0.80); $I^2 = 0\%$						
Test for overall effect: Z = 2.69 (P = 0.007)						
**1.1.3 Grade Ⅰ Fever**						
Ahmed 2016	1	81	4	84	13.2%	0.26 [0.03, 2.27]
Bhattar 2017	4	23	6	21	21.1%	0.61 [0.20, 1.86]
Demir 2022	6	67	16	70	52.6%	0.39 [0.16, 0.94]
Erturhan 2019	1	23	1	25	3.2%	1.09 [0.07, 16.39]
Mohey 2018	1	62	3	65	9.9%	0.35 [0.04, 3.27]
**Subtotal (95% CI)**		**256**		**265**	**100.0%**	**0.44 [0.24, 0.81]**
Total events	13		30			
Heterogeneity: $Chi^2 = 1.09$, df = 4 (P = 0.90); $I^2 = 0\%$						
Test for overall effect: Z = 2.64 (P = 0.008)						
**1.1.4 Grade Ⅰ Mucosal injury**						
Ahmed 2016	5	81	17	84	57.6%	0.31 [0.12, 0.79]
Bhattar 2017	3	23	8	21	28.9%	0.34 [0.10, 1.12]
Mohey 2018	1	62	4	65	13.5%	0.26 [0.03, 2.28]
**Subtotal (95% CI)**		**166**		**170**	**100.0%**	**0.31 [0.15, 0.63]**
Total events	9		29			
Heterogeneity: $Chi^2 = 0.05$, df = 2 (P = 0.97); $I^2 = 0\%$						
Test for overall effect: Z = 3.26 (P = 0.001)						
**1.1.5 Grade Ⅱ**						
Ahmed 2016	3	81	6	84	86.0%	0.52 [0.13, 2.00]
Erturhan 2019	2	23	1	25	14.0%	2.17 [0.21, 22.40]
**Subtotal (95% CI)**		**104**		**109**	**100.0%**	**0.75 [0.25, 2.29]**
Total events	5		7			
Heterogeneity: $Chi^2 = 1.09$, df = 1 (P = 0.30); $I^2 = 8\%$						
Test for overall effect: Z = 0.51 (P = 0.61)						
**1.1.6 GradeⅢa**						
Ahmed 2016	3	81	1	84	50.1%	3.11 [0.33, 29.30]
Mohey 2018	1	62	1	65	49.9%	1.05 [0.07, 16.40]
**Subtotal (95% CI)**		**143**		**149**	**100.0%**	**2.08 [0.39, 11.22]**
Total events	4		2			
Heterogeneity: $Chi^2 = 0.36$, df = 1 (P = 0.55); $I^2 = 0\%$						
Test for overall effect: Z = 0.85 (P = 0.39)						
**1.1.7 GradeⅢb and above**						
Aydın 2017	0	47	6	50	56.6%	0.08 [0.00, 1.41]
Bayar 2019	1	61	2	63	17.7%	0.52 [0.05, 5.55]
Erturhan 2019	1	23	3	25	25.8%	0.36 [0.04, 3.24]
**Subtotal (95% CI)**		**131**		**138**	**100.0%**	**0.23 [0.06, 0.89]**
Total events	2		11			
Heterogeneity: $Chi^2 = 1.11$, df = 2 (P = 0.57); $I^2 = 0\%$						
Test for overall effect: Z = 2.13 (P = 0.03)						

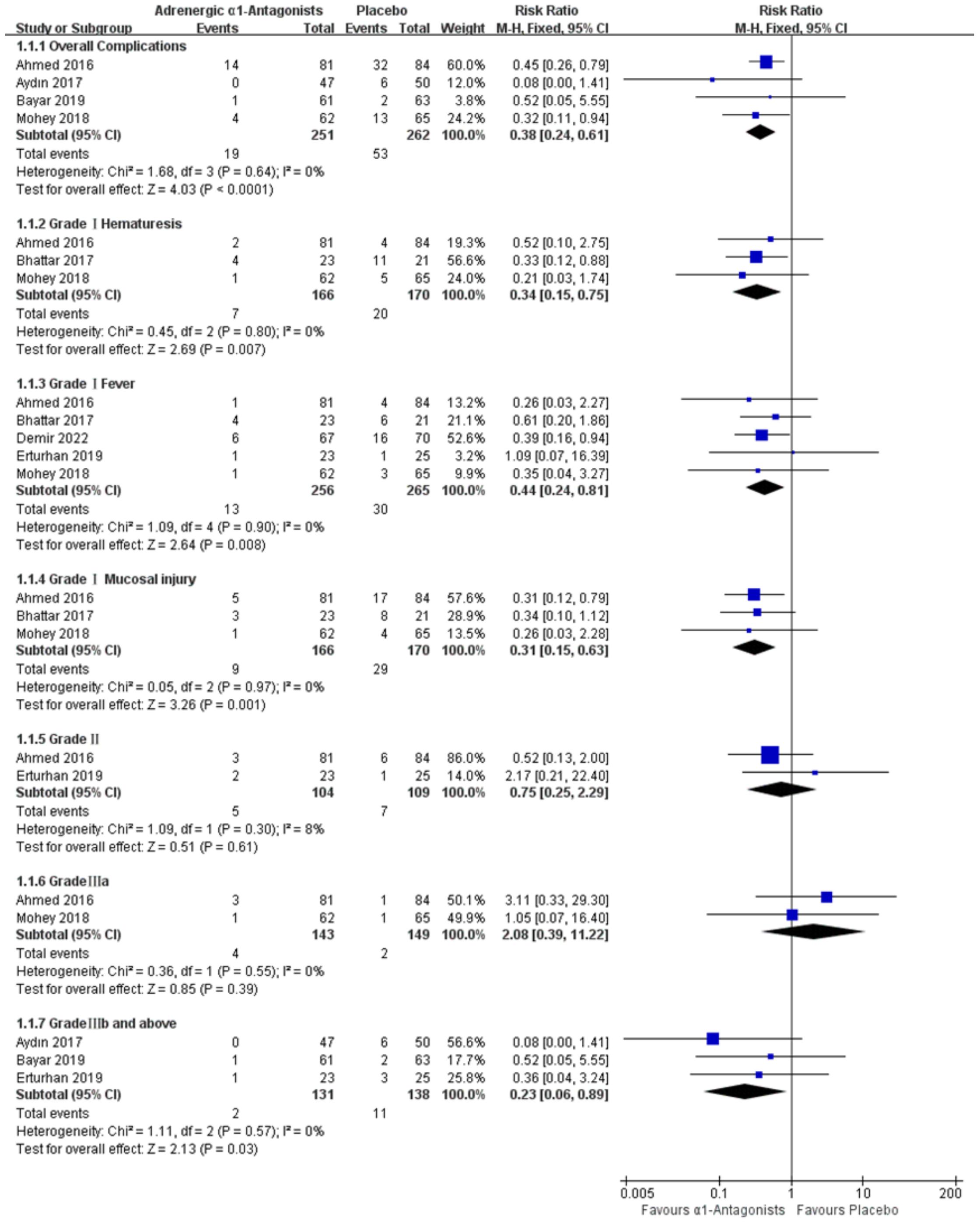

6 Successful access rate of AB

Study or Subgroup	Risk Difference	SE	Weight	Risk Difference IV, Random, 95% CI
Ahmed 2016	0.938272	0.02674	15.6%	0.94 [0.89, 0.99]
Aydın 2017	0.957447	0.029442	14.8%	0.96 [0.90, 1.02]
Bayar 2019	0.652174	0.099311	3.9%	0.65 [0.46, 0.85]
Bhattar 2017	0.73913	0.091561	4.4%	0.74 [0.56, 0.92]
Demir 2022	0.940299	0.028946	15.0%	0.94 [0.88, 1.00]
Erturhan 2019	0.967213	0.022801	16.7%	0.97 [0.92, 1.01]
McGee 2021	0.615385	0.134932	2.3%	0.62 [0.35, 0.88]
Mohey 2018	0.967742	0.022439	16.7%	0.97 [0.92, 1.01]
Morley 2020	0.88	0.045957	10.7%	0.88 [0.79, 0.97]
**Total (95% CI)**			**100.0%**	**0.92 [0.88, 0.96]**

Heterogeneity: Tau² = 0.00; Chi² = 23.93, df = 8 (P = 0.002); I² = 67%
Test for overall effect: Z = 42.29 (P < 0.00001)

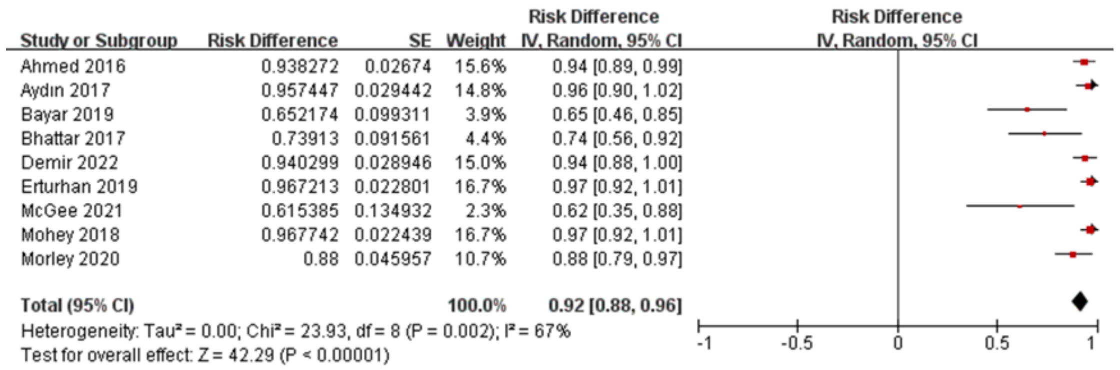

7 Successful access rate of placebo

Study or Subgroup	Risk Difference	SE	Weight	Risk Difference IV, Random, 95% CI
Ahmed 2016	0.821429	0.041788	12.5%	0.82 [0.74, 0.90]
Aydın 2017	0.76	0.060399	11.6%	0.76 [0.64, 0.88]
Bayar 2019	0.809524	0.049473	12.2%	0.81 [0.71, 0.91]
Bhattar 2017	0.285714	0.098581	9.5%	0.29 [0.09, 0.48]
Demir 2022	0.814286	0.04648	12.3%	0.81 [0.72, 0.91]
Erturhan 2019	0.44	0.099277	9.5%	0.44 [0.25, 0.63]
McGee 2021	0.388889	0.08125	10.5%	0.39 [0.23, 0.55]
Mohey 2018	0.8	0.049614	12.2%	0.80 [0.70, 0.90]
Morley 2020	0.653846	0.093301	9.8%	0.65 [0.47, 0.84]
**Total (95% CI)**			**100.0%**	**0.66 [0.55, 0.77]**

Heterogeneity: Tau² = 0.02; Chi² = 59.79, df = 8 (P < 0.00001); I² = 87%
Test for overall effect: Z = 11.77 (P < 0.00001)

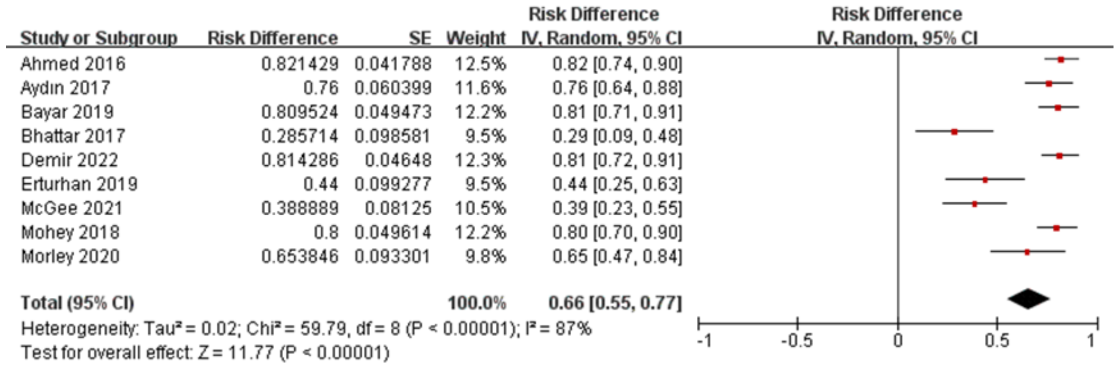

Supplement: Supplementary file 1 [file Datasheet1.pdf]
